# LFODet: Lightweight Few-Shot Object Detection with Meta-Learning in Remote Sensing Images

**DOI:** 10.3390/s26144371

**Published:** 2026-07-09

**Authors:** Haoran Wu, Xuan Fang, Haonan Xiong, Xiaomei Yang

**Affiliations:** 1 College of Electrical Engineering, Sichuan University, Chengdu 610065, China; hranw0831@stu.scu.edu.cn (H.W.); fangxuan@stu.scu.edu.cn (X.F.); xionghaonan1@stu.scu.edu.cn (H.X.); 2 Key Laboratory of Information and Automation Technology in Sichuan Province, Sichuan University, Chengdu 610065, China

**Keywords:** meta-learning, few-shot detection, object detection, lightweight convolutional neural network (CNN), remote sensing images (RSIs)

## Abstract

Balancing detection accuracy with model lightweightness remains a key challenge in remote sensing object detection. Although convolutional neural networks have improved performance, they typically require large-scale datasets, making few-shot detection of novel classes difficult. To tackle this, we propose LFODet, a lightweight few-shot object detection network based on meta-learning. It uses two parallel branches to rapidly adapt to novel classes with limited samples while maintaining performance on base classes. For efficient feature representation, we integrate Semantic Ghost Channel Attention (GCA) and Fine-Grained Ghost Spatial Attention (GSA) to enhance semantic discriminability and spatial detail preservation. Moreover, we leverage Ghost convolutions to reduce computational complexity. The model is trained in three stages: base-class pre-training, meta-learner optimization, and few-shot fine-tuning. Experiments on DIOR and NWPU VHR-10 demonstrate that LFODet achieves stable and balanced performance across various few-shot learning scenarios. As validated on these benchmark datasets, this work provides a practical solution for resource-constrained remote sensing applications requiring rapid adaptation to new targets.

## 1. Introduction

Remote sensing images (RSIs) have been widely used in numerous object detection tasks, such as vehicle surveillance, maritime rescue operations, and military reconnaissance [1]. However, compared with natural-image object detection, remote sensing object detection is more challenging because RSIs usually contain cluttered backgrounds, dense object distributions, large scale variations, and arbitrary viewing orientations. Recent advancements in deep learning, particularly convolutional neural networks (CNNs) and other neural network architectures, have significantly enhanced the accuracy of object detection in remote sensing images [2].

In addition, real-time object detection is required in many real-world applications, such as vehicle surveillance and maritime rescue operations. However, traditional object detection networks, such as Faster R-CNN [3] and AlexNet [4], are characterized by high memory consumption and significant computational complexity during inference. This is primarily attributed to the Region Proposal Network (RPN), which generates a large number of candidate regions. Consequently, these algorithms struggle to meet real-time requirements. Although lightweight models, such as the MobileNet series [5,6,7], the YOLO (You-Only-Look-Once) series [8,9,10], and the GhostNet series [11,12], offer a promising solution by significantly improving inference speed and enabling real-time data processing, they also face a critical challenge in balancing lightweightness and detection performance.

To improve the performance of lightweight models, several strategies have been proposed, such as strengthening feature representation and optimizing feature fusion strategies. Typically, feature representation can be enhanced by adding feature extraction modules and/or integrating feature enhancement modules into lightweight models. For example, the Light Global–Local Module has been proposed to extract global and local information, addressing the limitation of CNN-based lightweight models that overemphasize local information while neglecting global information [13]; the Selective Feature Enhancement Block has been proposed to selectively process a portion of feature maps that contribute more to semantic information extraction [14]. In addition, feature fusion networks are commonly used to improve the performance of lightweight models [15]. For example, a lightweight multi-scale feature fusion network has been proposed to integrate deep semantic and shallow spatial information [16], and a multi-scale fusion lightweight neck module has been introduced into YOLOv8 to fuse high-level and low-level feature information. However, most existing designs adopt uniform fusion strategies across different levels, which often leads to increased model complexity and feature redundancy. To address this issue, considering the unique characteristics of different network layers, we focus on a feature fusion network in this work. This network captures key semantic and spatial information while reducing feature redundancy by incorporating two lightweight attention modules, namely, Semantic Ghost Channel Attention (GCA) and Fine-Grained Ghost Spatial Attention (GSA), into a feature fusion network to capture fine-grained semantic and spatial information from different network layers.

However, these detection models typically require sufficient samples for training to perform well during the inference phase. Yet, acquiring sufficient training data is often infeasible due to high acquisition costs and the need for extensive human labor with expert domain knowledge for annotation. Moreover, some categories have only a few instances in real-world scenarios. Thus, there is a critical need to explore efficient object detection in the few-shot scenario. Recently, meta-learning, which enables models to acquire generalizable learning skills from prior task experience, has emerged as a viable solution for few-shot scenarios. One of the most classical meta-learning methods is Model-Agnostic Meta-Learning (MAML), which finds a proper model initialization and enables models to adapt quickly to the few samples of unseen classes. This versatility makes MAML particularly effective for few-shot object detection in remote sensing images [17]. While MAML has demonstrated effectiveness in classification tasks, it faces challenges in complex object detection problems due to the large parameter space [18]. Training the large parameter space of object detection models using few-shot novel-class samples tends to cause overfitting [19]. To address this issue, we propose a three-phase learning procedure based on the MAML strategy which incorporates a third Fine-Tuning stage to freeze a subset of network parameters. This approach reduces the parameter space, thereby improving the model’s performance and computational efficiency.

Therefore, we propose LFODet, a lightweight few-shot object detection network based on meta-learning, primarily motivated by the unique challenges in remote sensing images. The proposed network is characterized by its robustness and lightweight design. The key contributions of our work are as follows:

- We design a lightweight feature fusion network for remote sensing object detection. Instead of simply adding attention modules to the detector, LFODet uses GCA and GSA according to the different roles of multi-level features. GCA mainly enhances channel-level semantic information, while GSA helps preserve fine-grained spatial details. Together with Ghost-based operations, this design improves feature representation while maintaining a lightweight model structure.
- We develop a parameter-constrained three-phase learning procedure for lightweight few-shot detection. Instead of directly applying MAML to the whole detector, LFODet first learns a robust lightweight feature extractor on base classes, then optimizes a meta-learner with the feature extraction network frozen, and finally fine-tunes only the detection head using limited base and novel samples. This strategy constrains the trainable parameter space during few-shot adaptation and improves the stability of novel-class learning.
- We introduce a dual-branch inference strategy to balance novel-class adaptation and base-class retention, rather than simply merging two detection outputs. The base branch preserves base-class knowledge, while the novel branch adapts to few-shot novel classes. A confidence-based gating mechanism selects the more reliable branch using joint classification and objectness confidence, reducing unreliable predictions and alleviating base-class degradation after fine-tuning.
- Extensive comparative and ablation experiments on the DIOR and NWPU VHR-10 datasets demonstrate that LFODet can stably learn the features of novel classes as the number of shots increases, and it outperforms baseline methods under diverse few-shot conditions.

## 2. Related Work

### 2.1. Object Detection

Traditional object detection methods are broadly categorized into two-stage and one-stage approaches. Two-stage methods, such as RCNN [20], Fast RCNN [21], and Faster RCNN [22], involve generating region proposals, extracting regional features, and classifying objects. While these methods achieve high detection accuracy, they suffer from computational redundancy and low-quality region proposals [23].

In contrast, one-stage detectors like the YOLO series [8,9,10] and SSD (Single-Shot Detector) [24] adopt an end-to-end approach, eliminating the need for region proposal generation and thus achieving faster inference speeds with fewer parameters. For example, YOLOv3 [10] introduces a feature pyramid structure to capture multi-scale features, improving target information extraction. However, its feature fusion strategy remains simplistic, lacking the full utilization of feature refinement and contextual information.

This limitation becomes particularly evident in complex scenarios [25], such as remote sensing images, where targets vary in scale, shape, and occlusion, and the boundaries between targets and backgrounds are often ambiguous. It is difficult for simple feature fusion methods to make accurate judgments in these situations. Therefore, we propose an enhanced feature fusion network, emphasizing feature diversity to improve adaptability in complex scenes.

### 2.2. Lightweight Network

To develop application models for embedded devices, lightweight networks are widely adopted due to their reduced memory consumption and high computational efficiency. For example, ShuffleNet [26,27], a typical lightweight network, employs group convolution and channel shuffle operations to enhance information flow. GhostNet [11,12] simplifies the 1 × 1 convolution and incorporates Decoupled Fully Connected (DFC) attention to focus on fine-grained details. Despite their reduced computational complexity, these networks often struggle with tasks that require multi-scale and fine-grained feature extraction. In contrast, MobileNet [5,6,7] addresses these challenges by decomposing standard convolution into depthwise and pointwise convolutions and by introducing an inverted residual structure to preserve rich feature information. Thus, the MobileNet series have recently been widely used as an effective and lightweight solution for object detection. For example, MEANet adopts MobileNetv2 to extract the basic features of objects in optical remote sensing images [28], while Si-CA MobileNet was recently proposed for distracted driver detection [29].

To achieve a balance between performance and efficiency, MobileNetv3 [7] is adopted as the backbone in our model. However, lightweight networks often have limited feature representation capabilities, especially in object detection tasks that require simultaneous classification and localization. Thus, we add two attention modules, i.e., GCA and GSA, which focus on semantic and spatial fine-grained information, respectively, to enhance the model’s feature extraction capabilities. This design effectively addresses the inadequate feature extraction issue of lightweight detectors.

### 2.3. Few-Shot Learning

Few-shot learning approaches can be primarily categorized into three types: transfer learning, metric learning, and meta-learning. Transfer learning methods excel in localization tasks, with recent advancements incorporating supervised contrastive learning to enhance the compactness and variability between classes [30]. However, these methods often struggle with multi-object classification, especially when novel classes resemble base classes. Metric learning approaches address this issue by optimizing loss functions, such as triplet loss [31], to learn features with low intra-class and high inter-class distances, thereby encoding inputs of similar content into features with smaller differences. Recent advances like the Spatial-Channel State Space Model [32] and Transformer-based embedding learning [33] highlight the trend of using specialized modules (e.g., Mamba and Transformer) to enhance feature representation in few-shot object detection (FSOD). While these methods yield features with strong generalization capabilities, they require additional mechanisms to achieve accurate target localization in few-shot object detection scenarios.

Meta-learning methods enable models to ”learn how to learn”, facilitating rapid adaptation to new tasks. For example, Few-shot Detection with Reweighting (FSDW) [34] extracts global features from query images and embeds them into reweighting coefficients to adapt to novel classes. When there are N novel classes, the reweighting module will obtain N reweighting vectors, which can be used to extract important meta-features of the novel classes. FSODM [35] extends FSDW to multi-scale detection and demonstrates strong performance in remote sensing images. Meta-SSD [24] integrates a meta-learner with the SSD detector, enabling rapid parameter adaptation with few examples and proving the feasibility of combining SSD with meta-learning through experiments.

Since meta-learning tends to separate the foreground from the background rather than distinguishing between different classes, it can induce false positives [36], meaning that the predicted bounding box may not contain any actual target instances. However, object detectors need not only to identify whether a target category exists in the query set but also to distinguish among different classes. This is a challenge faced when using meta-learning approaches for object detection. To address this issue, we introduce a gating mechanism following the two parallel branches during inference to filter out false-positive bounding boxes.

By integrating meta-learning with lightweight networks and attention mechanisms, our LFODet mitigates the common overfitting issue in meta-learners when adapting to novel few-shot classes while simultaneously enhancing feature extraction capabilities to achieve robust performance.

## 3. Proposed Method

We propose LFODet, a lightweight object detection model that incorporates meta-learning and is tailored for remote sensing images. The primary objective of LFODet is to balance detection accuracy and model lightweightness, two prevalent issues in existing few-shot object detection algorithms.

### 3.1. Network Architecture

**Figure 1:**
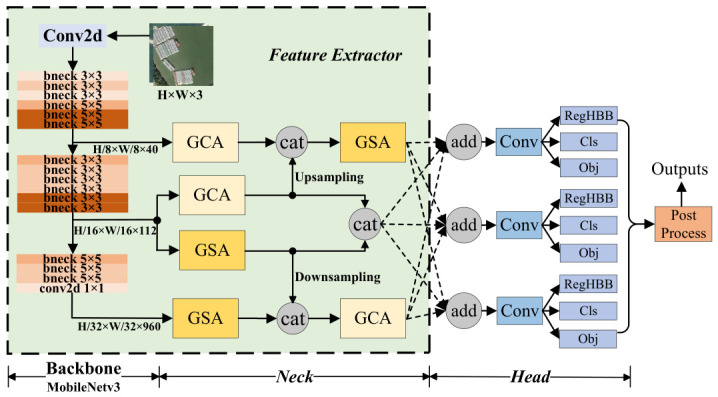
Structure of proposed lightweight network. The green-highlighted components in the backbone and neck represent our key modifications and improvements over the standard YOLO design.

The proposed model, LFODet, based on the YOLOv3 framework, comprises three main components: the backbone, the feature fusion neck, and the head, as illustrated in Figure 1. To reduce network complexity, MobileNetv3 replaces the backbone network of YOLOv3, which greatly reduces the number of network parameters. Additionally, the lightweight detection head of YOLOv3, characterized by fewer parameters, enables more efficient fine-tuning in few-shot scenarios. Adopting this classic architecture as a baseline provides a stable platform to rigorously validate the effectiveness of our core contributions.

#### 3.1.1. Backbone

The backbone of our model is based on MobileNetv3, which is employed to extract hierarchical features from remote sensing images. MobileNetv3 is selected for its computational efficiency and reduced memory consumption, enabling it to be deployed effectively in resource-constrained environments while still delivering strong performance. In this work, we retain the first 15 bnecks and one convolutional layer of MobileNetv3 while omitting the final pool and convolutional layers, as pool can lead to information loss. MobileNetv3 is divided into three blocks based on the 6th bneck, 12th bneck, and the final convolutional layer. The output features from these three levels are denoted by $f_{t}\in R^{h_{t}\times w_{t}\times c_{t}}\left(t=1,2,3\right)$, where $h_{t}$, $w_{t}$ and $c_{t}$ represent the height, width, and channel dimensions of the feature maps, respectively, and $\;c_{t}\in \left\{40,112,\left.960\right\}\right.$.

#### 3.1.2. Neck

**Figure 2:**
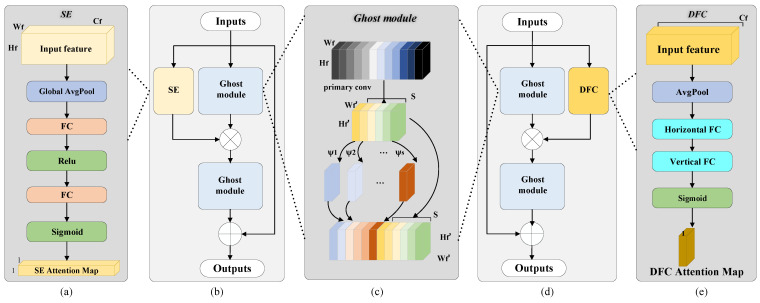
Structure of GCA and GSA modules. (**a**) SE module, (**b**) GCA module, (**c**) GHOST module, (**d**) GSA module, and (**e**) DFC module.

The neck component consists of two GhostBottleNecks with attention mechanisms, namely, GCA and GSA, as illustrated in Figure 2. These modules inherit the inverse residual structure of the GhostBottleNeck, which is composed of the Ghost submodule and attention mechanisms. The Ghost submodule is designed to reduce computational load and the number of parameters. However, many of the feature maps generated by the Ghost submodule are not directly related to useful features. To address this issue, as shown in Figure 2, the Squeeze-and-Excitation (SE) block [37], as a channel attention mechanism, is introduced into the GhostBottleNeck to form the GCA module. By leveraging the GhostBottleneck’s inherent redundancy reduction via Ghost operations, the GCA is well-suited for lightweight scenarios. Meanwhile, the DFC block, which serves as a long-distance spatial attention mechanism, is incorporated to form the GSA module. This structure is also referred to as the GhostBottleNeck in GhostNetv2 [11].

The GCA and GSA modules work together to enhance the model’s feature expression capabilities while maintaining low computational overhead. For low-level features from the backbone, which contain rich spatial fine-grained details but lack semantic information, they are first enhanced by the GCA module to improve channel semantics and then refined by the GSA module to enhance fine-grained spatial details. Conversely, for high-level features from the backbone, which are rich in semantic information but lack spatial fine-grained details, the GSA module first enhances spatial fine-grained details, followed by the GCA module for semantic compensation. For middle-level features, which possess both spatial fine-grained and semantic information, the GCA and GSA modules operate simultaneously to fully exploit both dimensions.

***(a) Ghost module:*** The Ghost module is the foundation of the GhostBottleNeck structure proposed in GhostNet [12], offering the advantage of low computational demand while retaining accuracy. As illustrated in Figure 2c, for an input feature map $I_{f}$ from one output branch of MobileNetv3, the Ghost submodule first generates intrinsic feature maps $F_{i}$ using a minimal number of traditional convolution operations. Subsequently, a series of simple, low-cost linear operations $\psi_{j}$ are applied to each intrinsic feature map to produce ghost feature maps $F_{g}$. It is important to note that the parameters required for these linear operations are significantly fewer than those needed for traditional convolution. Moreover, the ghost feature maps can effectively replace redundant features generated by conventional convolution. Finally, the intrinsic and ghost feature maps are concatenated along the channel dimension to produce an output feature map with the same number of channels as that of traditional convolution. Consequently, the Ghost module achieves lower total parameter counts and reduced computational complexity compared with ordinary convolution, without altering the size of the output feature maps.

For an input feature map $I_{f}\in R^{H_{f}\times W_{f}\times C_{f}}$, the Ghost module can be formulated as follows:

(1) $$\begin{array}{cc} \; & Ghost\left(I_{f}\right)=Concat\left(F_{i};F_{g}\right) \end{array}$$

(2) $$\begin{array}{cc} \; & F_{i}=ReLu\left(BN\left(Conv\left(I_{f},W_{1,1}\right)\right)\right) \end{array}$$

(3) $$\begin{array}{cc} \; & F_{g}=\psi_{j}\left(F_{i_{j}}\right)\;\;\;\;j=1,2,\ldots ,S, \end{array}$$

where Concat(·) is the concatenation operation along the channel dimension, Conv(·) is the convolution operation, BN(·) is batch normalization, ReLu(·) is the ReLu activation function, $W_{1,1}$ is a 1×1 convolutional kernel, and *S* represents the number of linear operations $\psi_{j}$.

According to the calculations by Han et al. [12], the Ghost module improves floating-point operations per second (FLOPs) and the compression ratio of parameters by a factor of *S* compared with ordinary convolution.

***(b) GCA module:*** The GCA module consists of a main branch and a residual branch, as shown in Figure 2b. The main branch includes one SE attention module and two Ghost modules. The SE attention module operates in parallel with the first Ghost module. Its role is to adaptively learn the relevance and importance of feature channels and assign corresponding channel weights. The feature maps extracted by the first Ghost module are then optimized through element-wise multiplication with these weights. This process selectively emphasizes significant features while suppressing irrelevant ones. Finally, the optimized feature maps are fed into the second Ghost module for channel compression and are connected to the residual branch to complete the module’s operation.

SE attention is a lightweight channel attention module, and its structure is shown in Figure 2a. Within the SE submodule, a global average pooling operation is applied to squeeze the input feature map $I_{f}$ of size $H_{f}\times W_{f}\times C_{f}$ into a $1\times 1\times C_{f}$ representation. In the excitation operation, two fully connected (FC) layers are used to learn the interdependencies between channels and to generate weight coefficients for each channel based on these learned dependencies. The formula for the SE block is as follows:

(4) $$W^{SE}=\sigma \left(FC_{2}\left(ReLu\left(FC_{1}\left(GAP\left(I_{f}\right)\right)\right)\right)\right)\in R^{1\times 1\times C_{f}},$$

where σ(·) is the sigmoid activation function; ${\mathrm{FC}}_{1}$ and ${\mathrm{FC}}_{2}$ are the first and second FC layers, respectively; and GAP(·) is the global average pooling. Finally, the feature map $I_{f}$ is reassigned the semantic weight through matrix multiplication as:

(5) $${\hat{F}}_{fc}=W_{1,1,k}^{SE}\times Ghost{\left(I_{f}\right)}_{i,j,k},$$

where $i=1,2\ldots ,H_{f}$, $j=1,2\ldots ,W_{f}$, $k=1,2\ldots ,C_{f}$, and ${\hat{F}}_{fc}$ is the semantic-attention weighted feature.

Subsequently, the weighted feature ${\hat{F}}_{fc}$ is fed into the second Ghost submodule, and its output features are concatenated with the residual branch. Finally, the GCA module outputs the enhanced feature $F_{GCA}$ as:

(6) $$F_{GCA}=Ghost\left({\hat{F}}_{fc}\right)+I_{f},$$

where the operator + is element-wise addition.

***(c) GSA Module:*** The GSA module integrates DFC attention and the Ghost module to further enhance fine-grained information, as shown in Figure 2d. Unlike the SE attention block, which focuses on channel-wise feature interactions, the DFC block emphasizes spatial attention calculations between different pixel blocks. Specifically, the DFC captures long-range fine-grained information by decoupling a traditional FC layer into two independent components: Horizontal FC and Vertical FC. This decoupling simplifies the computational complexity of the traditional FC layer, thereby meeting the lightweight requirements of the model.

In a standard FC layer, given a feature map $Z\in R^{H_{z}\times W_{z}\times C_{z}}$, we treat it as a set of $H_{z}\times W_{z}$ token vectors $z_{i}\in R^{C_{z}}$, and then $Z=\{z_{11},z_{12},\ldots ,z_{H_{z}W_{z}}\}$. The attention map $A=\{a_{11},a_{12},\ldots ,a_{H_{z}W_{z}}\}$ generated by the FC layer is defined as

(7) $$a_{hw}=\sum_{h^{\prime },w^{\prime }}F_{hw,h^{\prime }w^{\prime }}⨀z_{h^{\prime }w^{\prime }},$$

where ⨀ denotes element-wise multiplication and $F_{\cdot ,\cdot }$ denotes the learnable weight parameters in the FC layer. However, the computational complexity of the attention map is $O(H_{z}^{2}W_{z}^{2})$.

To reduce computational costs, the DFC attention block decomposes the FC layer in Equation (7) into separate vertical and horizontal FC layers, as shown in Figure 2e. We first apply average pooling as a downsampling operation to the input feature map $I_{f}\in R^{H_{f}\times W_{f}\times C_{f}}$, yielding a smaller feature map $Z\in R^{H_{z}\times W_{z}\times C_{z}}$. This downsampled feature map *Z* is then used to aggregate features along the vertical and horizontal directions, formulated as follows:

(8) $$\begin{array}{cc} a_{hw}^{\prime } & =\sum_{h^{\prime }}^{H_{z}}F_{h,h^{\prime }w}^{H}⨀z_{h^{\prime }w},\;\;\;h=1,2,\ldots ,H_{z},w=1,2,\ldots ,W_{z}\\ a_{hw} & =\sum_{w^{\prime }}^{W_{z}}F_{w,hw^{\prime }}^{W}⨀a_{hw^{\prime }}^{\prime },\;\;\;h=1,2,\ldots ,H_{z},w=1,2,\ldots ,W_{z}\\ F_{D} & =\sigma (a_{hw}) \end{array}$$

where $F_{\cdot ,\cdot }^{H}$ and $F_{\cdot ,\cdot }^{W}$ denote the learnable weights and $F_{D}$ represents the aggregated feature map. By decoupling the horizontal and vertical transformations, the computational complexity of the DFC block is reduced to $O(H_{z}^{2}W_{z}+H_{z}W_{z}^{2})$.

Subsequently, the feature map $F_{D}$ is upsampled via bilinear interpolation to restore its original size $H_{f}\times W_{f}\times C_{f}$, and it serves as the attention feature to weight the feature map from the first Ghost submodule. In this way, important spatial features are enhanced while unimportant ones are suppressed. The spatial attention weighted features ${\hat{F}}_{fs}$ can be expressed as follows:

(9) $${\hat{F}}_{fs}=Up\left(F_{D}\right)\times Ghost\left(I_{f}\right),$$

where Up(·) denotes the upsampling operation.

Similar to the GCA module, the GSA module processes ${\hat{F}}_{fs}$ through the second Ghost submodule and incorporates a residual connection. The enhanced feature output by the GSA module is then expressed as:

(10) $$F_{GSA}=Ghost\left({\hat{F}}_{fs}\right)+I_{f},$$

where $F_{GSA}$ is the enhanced feature produced by the GSA module.

#### 3.1.3. Head

To ensure that input features of different resolutions have an equal impact on the output features, the model head is the sum of feature maps from each branch, including those processed through upsampling and downsampling operations, some of which are obtained after upsampling or downsampling operations. This fusion operation ensures that each branch contributes effectively to the final output. The model ultimately calculates the bounding-box position regression, target category score, and confidence score. This multi-branch approach enables the model to handle objects of varying sizes and aspect ratios, thereby enhancing its performance on remote sensing images with diverse levels of detail.

### 3.2. Three-Phase Learning Procedure

To achieve rapid adaptation on few-shot novel-class samples, we divide the learning process into three stages, i.e., Base Training, Meta-Training, and Fine-Tuning. Initially, during the Base Training stage, the network is trained on a large number of samples from the base classes to learn general image features. This stage employs random initialization of network parameters and aims to develop a robust feature extractor capable of handling diverse image characteristics.

Next, the Meta-Training stage focuses on capturing the commonalities between the base-class and novel-class features and identifying the learning rules for adapting to novel classes. Using base-class samples, a meta-learner is trained to optimize the head network parameters. The goal is to establish a meta-learning framework that can rapidly adapt to new tasks with limited data.

Finally, in the Fine-Tuning stage, the model is re-trained on limited samples that include both base-class ($D_{base}$) and novel-class ($D_{novel}$) data. The parameters of the head network are only updated to fine-tune the model for the specific characteristics of the novel classes, whereas the weights of the feature extraction network learned in the first two training stages are frozen. This stage aims to achieve the ultimate goal of rapidly adapting to few-shot novel-class samples. Throughout all stages, the entire network is optimized in an end-to-end manner using the same objective function.

#### 3.2.1. Base Training

To obtain a highly robust feature extraction network F, a large number of base-class images are input into the model. The output feature maps after extraction are denoted by $f_{t}=F\left(x\right)\in R^{\frac{H}{D_{t}}\times \frac{W}{D_{t}}\times C_{t}}\left(t=1,2,3\right)$, where stride $D_{t}\in \left\{8,\left.16,32\right\}\right.$. In order to enhance the network’s feature representation capacity, the number of channels is increased, so that the channel $C_{t}\in \left\{256,\left.512,1024\right\}\right.$.

The extracted feature maps $f_{t}$ are fed into the head of the LFODet model, which outputs a tensor of dimensions $g\times g\times N\times \left(\left(t_{x},t_{y},t_{w},t_{h}\right)+{obj}_{score}+{class}_{score}\right)$; this tensor contains predicted bounding-box information, where g×g represents the size of the grid cells, *N* represents the number of predicted bounding boxes for each grid cell, $\left(t_{x},t_{y},t_{w},t_{h}\right)$ represent the predicted offsets of the bounding boxes and the ratio coefficients relative to the width and height of the anchor boxes, ${obj}_{score}$ is the confidence score indicating the presence of an object, ${class}_{score}$ is the probability of the object’s category. To optimize the model, three loss components are defined: location loss, confidence loss, and category loss. Specifically, the location loss measures the accuracy of the predicted bounding boxes compared with the ground-truth boxes, the confidence loss quantifies the difference between the predicted confidence score of a bounding box and the ground truth, and the category loss helps the model learn to correctly classify objects into predefined classes. By summing these three loss terms, the total loss is constructed as follows:

(11) $$\begin{array}{cc} \; & Loss={Loss}_{loc}+{Loss}_{conf}+{Loss}_{cls}, \end{array}$$

where ${Loss}_{loc}$ is the location loss, ${Loss}_{conf}$ is the confidence loss, and ${Loss}_{cls}$ is the category loss.

For ${Loss}_{loc}$, as shown in Equation (12), we employ the Complete Intersection Over Union (CIOU) regression [38] to measure the accuracy of the predicted bounding boxes. This method considers the overlapping area, center distance, and shape similarity between the predicted and ground-truth bounding boxes. The CIOU loss is used as a key component in calculating the location loss.

(12) $$\begin{array}{cc} \; & {Loss}_{loc}=L_{mask}\times b_{scale}\times L_{CIOU}, \end{array}$$

where $L_{mask}$ indicates the presence of an object, $b_{scale}$ balances the loss effect of large and small targets by adjusting the ratio of the area of the ground-truth box to the total area of the image, and $L_{CIOU}$ represents the CIOU loss [38], calculated as:

(13) $$L_{CIOU}=1-IOU+\frac{\rho^{2}\left(A,B\right)}{C^{2}}-\alpha \times V$$

where

$$IOU=\frac{A\cap B}{A\cup B},\;\;\;\;\;\;\;\;\;\;\;\;\;\;\;\;\;\;\alpha =\left\{\begin{array}{c} 0\;\;\;\;\;\;\;IOU<0.5,\\ \frac{V}{(1-IOU)+V}\;IOU\geq 0.5, \end{array}\right.$$

and

(14) $$V=\frac{4}{\pi^{2}}\left(arctan\frac{w_{g}}{h_{g}}-arctan\frac{w_{p}}{h_{p}}\right),$$

where *A* and *B* represent the ground-truth and predicted boxes, respectively; $w_{g}$ and $h_{g}$ are the width and height of the ground-truth box, respectively; $w_{p}$ and $h_{p}$ are the width and height of the prediction box, respectively; IOU quantifies the overlap between the two boxes; $\rho^{2}\left(A,B\right)$ is the squared Euclidean distance between the centers of the ground-truth and predicted boxes; $C^{2}$ is the smallest bounding box’s diagonal length; α acts as the trade-off parameter; and *V* denotes the consistency of the aspect ratio used to further adjust the loss function.

For ${Loss}_{conf}$, to address the imbalance between the number of positive and negative samples, we introduce the Focal Loss [39] in the confidence loss. This loss function reduces the weight of easy (both positive and negative) samples while increasing the influence of hard (both positive and negative) samples. The formulation is as follows:

(15) $${Loss}_{conf}=L_{mask}\times FL\left(p_{t}\right)+L_{nomask}\times FL\left(p_{t}\right),$$

where $FL\left(p_{t}\right)$ represents Focal Loss, defined as

(16) $$FL\left(p_{t}\right)=-\alpha {\left(1-p_{t}\right)}^{\gamma }log\left(p_{t}\right),$$

where $p_{t}$ is the predicted probability of the target class, and α and γ are hyperparameters that control the balance between positive and negative samples and adjust the focus on hard versus easy samples.

For $Loss_{cls}$, we utilize the Binary Cross-Entropy (BCE) loss to measure the discrepancy between the predicted probability vector p and the ground-truth label vector g in *J*-dimensional category space, formulated as follows:

(17) $${Loss}_{cls}=L_{mask}\times BCE\left(p,g\right),$$

where $BCE\left(p,g\right)$ computes the cross-entropy value, defined by

(18) $$BCE\left(p,g\right)=-\frac{1}{J}\sum_{t=1}^{J}\left(g_{t}log\left(p_{t}\right)+\left(1-g_{t}\right)log\left(1-p_{t}\right)\right),$$

where $p_{t}$ denotes the predicted probability for the t-th target class and $g_{t}$ denotes its corresponding ground-truth label.

Finally, by minimizing the total Loss in (11), backpropagation updates the parameters of the entire network, including the backbone, neck, and head components.

#### 3.2.2. Meta-Training

To fully leverage the knowledge acquired during Base Training and reduce the parameter space, we freeze the feature extraction network during Meta-Training. The introduced meta-learner, which shares the same structure as the head network, is trained using the MAML method. Unlike traditional machine learning, where datasets are typically divided into classes and models are trained to classify these classes, meta-learning divides the datasets into multiple tasks $T_{i}$. Each task consists of a support set $S_{i}$ and a query set $Q_{i}$, with 2K samples per class; i.e., $T_{i}=S_{i},Q_{i}$. This approach enables the model to rapidly adapt to different tasks, rather than merely learning fixed classification rules from a single task.

The support set plays a crucial role in calculating the gradient and guiding the model to learn how to extract useful features from limited samples. However, it does not directly update the final parameters of the LFODet model. Instead, the support set is used solely for rapid adaptation within the model without affecting the global model update. This process is commonly referred to as the inner loop. The rapid adaptation is formulated as follows:

(19) $$\begin{array}{cc} \; & \theta^{\prime }=\theta -lr_{in}\nabla_{\theta }Loss_{T_{i}}\left(f_{i}^{s};\theta \right), \end{array}$$

where θ is the current model parameter, $\theta^{\prime }$ is the updated parameter after the gradient update, $lr_{in}$ is the inner-loop learning rate that controls the step size of the gradient descent, $f_{i}^{s}$ is the feature extraction output from the support set $S_{i}$, and $\nabla_{\theta }Loss_{T_{i}}\left(f_{i}^{s};\theta \right)$ is the gradient of the loss function with respect to the model parameters on the support set $S_{i}$.

The role of the query set is to assess the LFODet model’s generalization ability. Using the updated parameters $\theta^{\prime }$, the LFODet model makes predictions on the query set and calculates the corresponding loss function. This process is known as the outer loop, as shown below:

(20) $$\begin{array}{cc} \; & \min_{\theta }\sum_{T_{i}}^{M}Loss_{T_{i}}\left(f_{i}^{q};\theta^{\prime }\right), \end{array}$$

where $f_{i}^{q}$ is the feature extraction output of the query set $Q_{i}$ and $\sum_{T_{i}}^{M}Loss_{T_{i}}\left(f_{i}^{q};\theta^{\prime }\right)$ represents the loss for all tasks. This loss term is designed to enable the model to better adapt to the demands of diverse tasks. Within the outer loop, the model parameters θ are updated as follows:

(21) $$\theta =\theta -lr_{out}\nabla_{\theta }\sum_{T_{i}}^{M}Loss_{T_{i}}\left(f_{i}^{q};\theta^{\prime }\right),$$

where $lr_{out}$ denotes the learning rate for the outer loop.

The detailed Meta-Training algorithm is summarized in Algorithm 1. This iterative process enables the model to be trained and optimized across diverse tasks, thereby enhancing its ability to perform well on new tasks and rapidly adapt to limited samples.
**Algorithm 1** Meta-learning procedure.**Require:** Model parameters θ pre-trained in the base training stage;     Freeze the feature extraction network;     Support set $S_{i}$ and query set $Q_{i}$ for each task $T_{i}$;     Inner-loop learning rate $lr_{\mathrm{in}}$ and outer-loop learning rate $lr_{\mathrm{out}}$;     Training epochs: epochs; **Ensure:** Updated model parameters θ;
1:**for** i < epochs **do**2:     Sample a batch of tasks $T_{i}$;3:     **for** all $T_{i}$ **do**4:      Compute the gradient $\nabla_{\theta }Loss_{T_{i}}\left(f_{i}^{s};\theta \right)$ with respect to K examples;5:      Update parameters via gradient descent following Equation (19);6:     **end for**7:     Update parameters θ following Equation (21)8:**end for**

#### 3.2.3. Fine-Tuning

According to the research study by Chen et al. [40], limited training data can lead to model overfitting, especially when dealing with few-shot novel classes. This overfitting can result in false positives, particularly for objects that are visually similar. To alleviate these problems, we fine-tune the head network while freezing the feature extraction network, whose parameters are inherited from the Base and Meta-Learning stages, and augment the training data by randomly sampling K samples from each base class. This approach helps increase the diversity of training classes and reduces the likelihood of overfitting to the limited novel-class samples.

### 3.3. Inference

**Figure 3:**
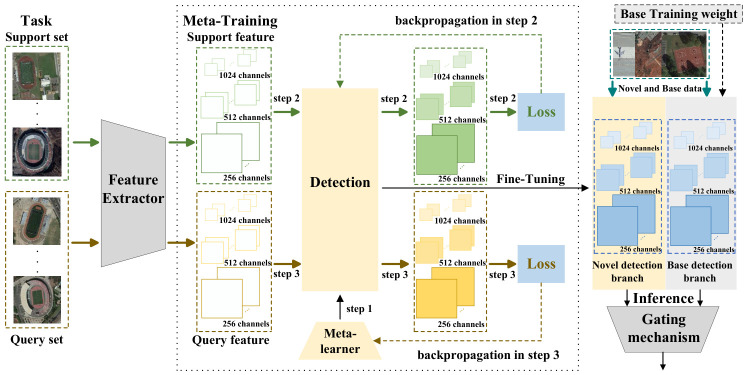
Meta-Training and Fine-Tuning stages.

To maintain base-class detection performance, we introduce a base detection branch initialized with the parameters obtained from the Base Training stage and integrate it with the novel detection branch, as shown in Figure 3. In dual-branch inference, the main challenge is to combine the base and novel branches without weakening their respective roles. Since the two branches are optimized under different data conditions, directly blending their outputs may reduce the response of the more reliable branch, while introducing an additional learnable fusion module may increase the risk of overfitting in the few-shot setting. Therefore, we adopt a gating mechanism. This gating calculates a total score based on classification and confidence [41] to filter out the branch with the higher score. The formula for this gating strategy is defined as follows:

(22) $$O_{D}\left(i,j\right)=\left\{\begin{array}{c} O_{B}\left(i,j\right)\;\;\;\;\;if\;\;\;\left\{\max\left(O_{B}^{cls}\right)*O_{B}^{obj}\right\}\left(i,j\right)\geq \left\{\max\left(O_{N}^{cls}\right)*O_{N}^{obj}\right\}\left(i,j\right)\\ O_{N}\left(i,j\right)\;\;\;\;\;\;otherwise, \end{array}\right.$$

where $O_{B}$ and $O_{N}$ denote the outputs of the base-class detector and the current-class detector, respectively, and $\left(i,j\right)$ denote the spatial grid coordinates.

The proposed gating mechanism fits the lightweight few-shot setting because it selects between the two branches without introducing additional trainable parameters or requiring extra data for learning a fusion function. This confidence-based selection also preserves the specialization of the base and novel branches, allowing LFODet to balance base-class retention and novel-class adaptation.

Nevertheless, this strategy also has potential limitations. Since the gating mechanism performs hard branch selection according to confidence scores, it may fail when the confidence scores of the two branches are not well-calibrated. In addition, hard selection cannot fully exploit complementary information from both branches when both branches provide partially useful predictions.

## 4. Experiments and Results

### 4.1. Experimental Platform and Settings

The experiments in this study were conducted using the PyTorch 1.7.0 deep learning framework on a computer equipped with an NVIDIA GeForce RTX 3060 GPU. Detailed training configurations are provided in Table 1. To improve reproducibility, the source code, configuration files, few-shot split files, sampling scripts, and instructions for obtaining the pre-trained weights have been made publicly available in our GitHub repository (https://github.com/xxxxfang/LFODet (accessed on 6 July 2026)).

### 4.2. Dataset and Implementation Details

The proposed model, LFODet, is validated on the DIOR [42] and NWPU VHR-10 [43] datasets. The DIOR dataset is a high-resolution aerial remote sensing dataset widely used for object detection tasks. It consists of 23,463 images with 192,472 annotated instances across 20 object classes, with each image having a resolution of 800 × 800 pixels. The dataset is divided into training, validation, and test sets in a 2:1:1 ratio. To adapt to the few-shot scenario, the dataset is further partitioned into 15 base classes (airport, basketball court, bridge, chimney, dam, expressway service area, expressway toll station, golf field, ground track field, harbor, overpass, ship, stadium, storage tank, and vehicle) and 5 novel classes (airplane, baseball field, tennis court, train station, and windmill).

The NWPU VHR-10 dataset contains 800 very-high-resolution (VHR) optical remote sensing images, with 10 classes. A total of 650 samples in the dataset are treated as positive images, which means that they contain at least one target, while 150 samples are treated as negative images, which means that they do not contain any targets. In the few-shot scenario, we choose seven classes (ship, storage tank, tennis court, basketball court, ground track field, harbor, and vehicle) as base classes and the remaining three classes (airplane, baseball diamond, and bridge) as novel classes.

In the construction of the few-shot training sets, we adopted a class-wise random sampling protocol. Specifically, for each dataset and each K-shot setting, candidate samples were first organized according to their class labels, and K annotated instances were randomly selected from each target class to construct the corresponding few-shot training list. For the Fine-Tuning stage, few-shot lists containing both base and novel classes were constructed using the same class-wise K-shot sampling rule. For the Meta-Training stage, samples were organized into support/query sets according to the same task-level sampling protocol. The sampling script used to generate these few-shot lists has been released in our GitHub repository to document the exact sampling procedure.

Specifically, during the Base Training stage, samples from the base classes in the training set are used for model initialization. In the Meta-Training stage, base-class samples are randomly selected from both the training and validation sets. For the Fine-Tuning stage, K=3,5 and 10 instances from the base and novel classes are randomly sampled from the training and validation sets. The model is evaluated on a test set containing all classes.

To enhance the model’s generalization ability in few-shot scenarios and mitigate overfitting, we adopt the Mixup data augmentation strategy [44]. Mixup generates new training samples by linearly interpolating two randomly selected training images, thereby expanding the distribution range of the training data.

In the Fine-Tuning stage, we incorporate a learning rate warm-up and progressive decay strategy to enhance training stability. Specifically, for different K-shot settings, linear warm-up is configured for distinct training epochs, where the learning rate increases linearly from zero to the preset value. After warm-up, the learning rate is reduced to 20% of its original value at a specific training epoch.

### 4.3. Evaluation Metrics

Performance is evaluated using the Average Precision (AP) metric, a widely used indicator in object detection for single-category assessment. The Precision–Recall(PR) curve is generated with Precision (P) on the vertical axis and Recall (R) on the horizontal axis, and AP is computed as the area under the PR curve. Precision and Recall are calculated using true positives (TP), false positives (FP), false negatives (FN), and true negatives (TN), with an Intersection over Union (IoU) threshold set to 0.5. Additionally, mean Average Precision (mAP) represents the average AP across all classes, novel Average Precision (nAP) denotes the average AP across novel classes, and base Average Precision (bAP) indicates the average AP across base classes. The related equations are as follows:

(23) $$\begin{array}{cccc} \; & AP=\int_{0}^{1}P(R)dR\; & R=\frac{TP}{TP+FN}\;\;\;\;\;\;\;\;\; & P=\frac{TP}{TP+FP}\\ \; & mAP=\frac{1}{C_{a}}\sum_{i=0}^{C_{a}}AP_{i}\;\; & bAP=\frac{1}{C_{b}}\sum_{i=0}^{C_{b}}AP_{i}\;\; & nAP=\frac{1}{C_{n}}\sum_{i=0}^{C_{n}}AP_{i} \end{array}$$

where $C_{a}$, $C_{b}$ and $C_{n}$ denote the number of all classes, base classes, and novel classes, respectively. Please refer to Section 4.2 for the corresponding class counts of the two datasets used in our experiments. Model parameters (Params; M, in millions) are used to evaluate model size, while floating-point operations (FLOPs; G, in billions) assess computational complexity, which are computed using the PyTorch-based thop profiler (version 0.1.1.post2209072238). In addition, inference speed is reported in frames per second (FPS), which is calculated as the reciprocal of the average detection time per image. The reported detection time includes image preprocessing, model forward propagation, bounding-box decoding, and non-maximum suppression (NMS) but excludes image loading, file writing, and AP calculation.

### 4.4. Results and Comparisons

To further validate the effectiveness of the proposed method, we conducted comparisons with the following approaches: (1) the first-order variant of the MAML method [45]; (2) the standard Fine-Tuning method [18]; (3) the Two-Stage Fine-Tuning method (TFA) [46]; (4) the few-shot detection method BC-YOLO [41]; (5) BC-YOLO’s deformation, BC-YOLO* [41]; and (6) a meta-learning-based method, AOFS [47], for arbitrary-oriented FSOD. To ensure a fair comparison, all compared methods were trained and evaluated using the same base-/novel-class splits, the same K-shot sampled instance lists, and the same test sets under each few-shot setting. Regarding the backbone settings, Table 2 explicitly reports the backbone used by each method. MAML, Fine-Tuning, TFA, and BC-YOLO* adopt MobileNetV3 as the backbone, while BC-YOLO and AOFS retain their original backbone settings, namely, Darknet-53 and CSPDarknet-53, respectively. This is because these backbones are part of their original method designs. For training settings, the same basic data organization and few-shot sampling protocol were used for all methods, while method-specific components were retained when required by the original methods.

We conducted repeated experiments to improve the statistical reliability of the few-shot results. Specifically, the first stage, Base Training, and the second stage, Meta-Training, were conducted once, while the third stage, Fine-Tuning, and evaluation were repeated over five independently sampled few-shot splits under each K-shot setting. The comparative results are reported as the mean and standard deviation over the five trials.

**Figure 4:**
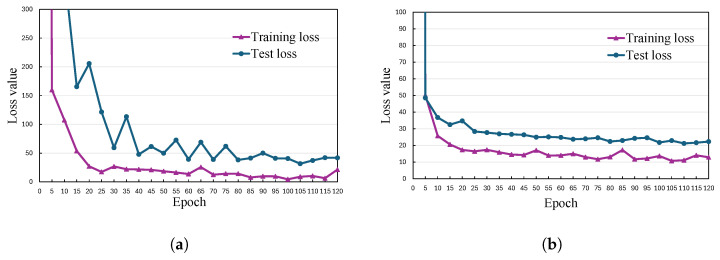
Training and test loss curves on the DIOR and NWPU VHR-10 datasets during the Base Training of LFODet. (**a**) Base Training loss on DIOR dataset. (**b**) Base Training loss on NWPU VHR-10 dataset.

Figure 4 presents the loss curves of the LFODet method during the Base Training phase on the DIOR and NWPU VHR-10 datasets. On both datasets, the training and test losses consistently decreased and eventually stabilized.

**Figure 5:**
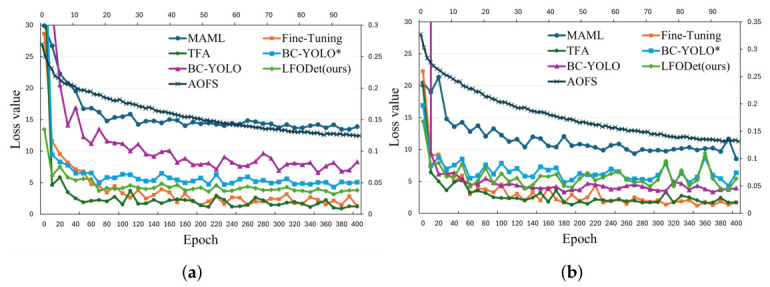
Training loss curves on the DIOR and NWPU VHR-10 datasets during the 10-shot Fine-Tuning of LFODet and few-shot training loss of the other baseline methods. (**a**) Training loss on DIOR dataset. (**b**) Training loss on NWPU VHR-10 dataset. Note: Main coordinate system (left Y-axis, bottom X-axis) displays the results of MAML, Fine-Tuning, TFA, BC-YOLO *, BC-YOLO, and LFODet, with the X-axis representing epochs (1–400) and the Y-axis representing loss (0–30). Sub-coordinate system (right Y-axis, top X-axis) displays AOFS method, where the X-axis represents epochs (1–100) and the Y-axis represents loss (0–0.35).

Figure 5 also illustrates the 10-shot training loss curves of LFODet during the Fine-Tuning phase and the few-shot training loss curves of other baseline methods on the DIOR and NWPU VHR-10 datasets. Due to the significantly lower performance indicators of the AOFS method compared with the other baseline methods, in order to maintain chart clarity, we present it on the secondary axis. In the experiment, the original papers’ parameter settings were fully followed, and model training reached a convergence state. LFODet demonstrates a faster convergence rate and more stable loss reduction compared with the baselines.

#### 4.4.1. Numerical Evaluation

The quantitative results in Table 2 reveal that LFODet outperforms baseline methods on the DIOR and NWPU VHR-10 datasets under all few-shot settings (3 shots, 5 shots, and 10 shots), achieving the highest mAP in each case. The results of the proposed model, LFODet, are based on the third stage, Fine-Tuning.

Especially on the DIOR dataset, which includes diverse object classes, LFODet performs well in both base- and novel-class detection. Where the six baseline models exhibit issues such as overfitting to novel classes, neglecting base classes, or struggling with complex scenes, LFODet maintains a balanced and stable detection performance. In particular, while baseline methods may suffer from poor generalization or weak feature adaptation, especially under the 3-shot and 5-shot settings, LFODet continues to provide reliable predictions for both frequently occurring and novel targets. Even on the challenging NWPU VHR-10 dataset, which contains VHR optical images and fewer classes, LFODet further validates its effectiveness in handling detailed visual information with limited training data.

In the 3-shot setting on the NWPU VHR-10 dataset, LFODet shows stable performance on base classes and achieves the highest overall mAP. However, compared with baseline methods such as Fine-Tuning and TFA, its nAP still shows a certain gap. This result suggests a possible trade-off in few-shot detection: preserving base-class knowledge can improve overall stability but may also limit the model’s adaptation to novel classes to some extent. Fine-Tuning and other baseline methods adapt to novel classes by updating model parameters more extensively, which may improve novel-class performance but can also lead to more severe catastrophic forgetting on base classes. In contrast, LFODet is designed to balance novel-class adaptation and base-class preservation through its lightweight architecture, feature fusion strategy, and dual-branch inference design, which is reflected in its overall mAP performance.

In summary, these results validate the effectiveness of LFODet in handling data scarcity, class imbalance, and target complexity. LFODet achieves stable few-shot detection performance while maintaining base-class recognition ability, highlighting its potential for practical remote sensing applications.

Table 3 reports the per-class AP results of novel classes on the DIOR dataset. Compared with the aggregated nAP metric in Table 2, the per-class results reveal the detection difficulty of different novel categories more explicitly. Overall, the per-class results provide a more detailed category-level view of LFODet under few-shot settings. Although the AP values remain uneven across different novel categories, LFODet achieves competitive nAP and shows improved category-level performance as the number of shots increases. The results also show that AP varies across categories, which may be related to differences in object scale, appearance variation, background complexity, and the limited number of support samples. As the number of shots increases, the model generally obtains more stable category-level performance, further demonstrating the benefit of additional few-shot annotations for novel-class adaptation.

**Figure 6:**
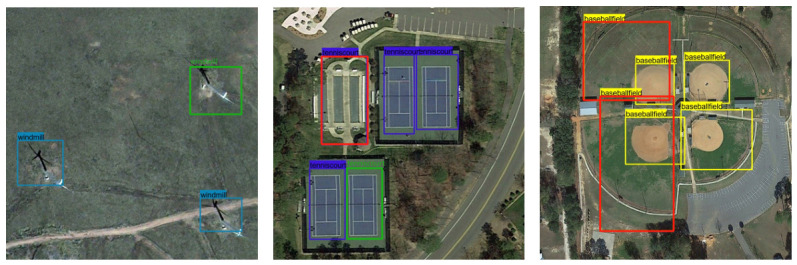
Visualization of failure cases on the DIOR dataset under the 10-shot setting. Note: Red boxes indicate false detections, while green boxes indicate missed detections.

Meanwhile, LFODet exhibits typical failure cases in the experiments, as shown in Figure 6. The causes can be summarized as follows: (1) Low Background Discrimination: Repeated detection of anchor frames may occur due to insufficient background differentiation. This limitation on the number of detectable anchor frames can lead to missed detections of similar, unobserved targets. (2) Target Similarity: Objects with similar sizes and shapes, such as tennis courts and other sports facilities, are prone to confusion. This issue also affects other object pairs, such as cars vs. ships or bridges vs. overpasses. (3) Poor Accuracy for Large Targets: Compared with more sophisticated CNN models, our lightweight network exhibits less efficient deep semantic feature extraction, particularly for large targets with complex textures, such as golf courses and airports.

#### 4.4.2. Visualization Evaluation

**Figure 7:**
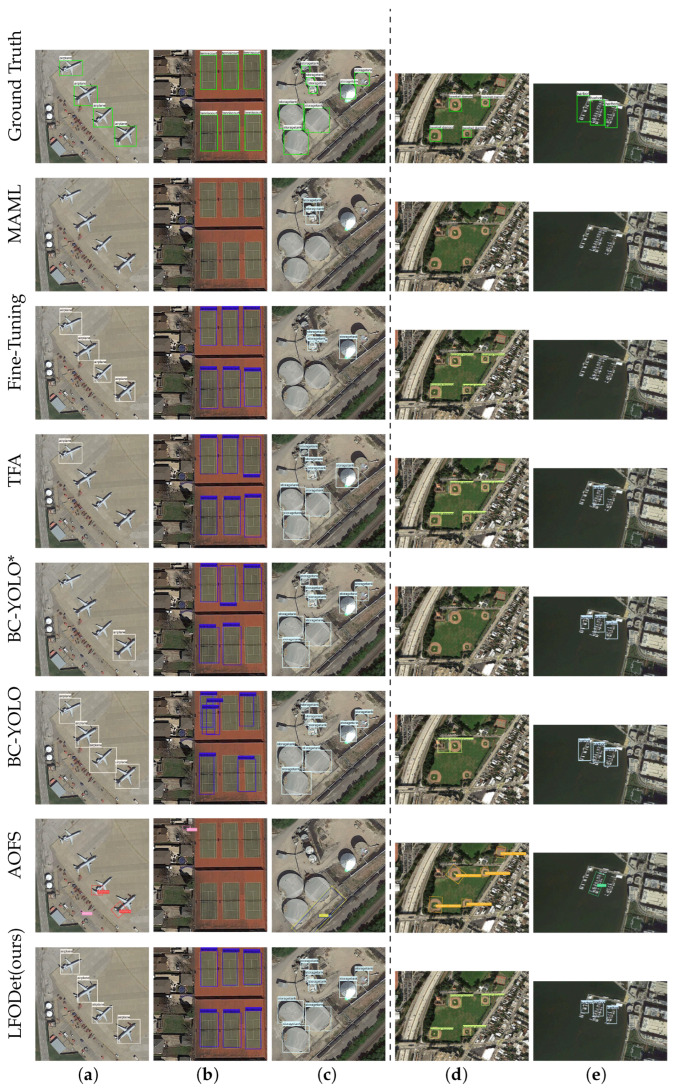
Object detection visualization under 10-shot setting for all methods. (**a**) Airplane from DIOR. (**b**) Tennis court from DIOR. (**c**) Storage tank from DIOR. (**d**) Baseball diamond from NWPU VHR-10. (**e**) Harbor from NWPU VHR-10. * Ground truth of AOFS, based on the rotation transformation result of the first row target box (rotation diagram omitted due to space limitations).

In order to make the experimental results more intuitive, we selected representative novel and base classes from the DIOR and NWPU VHR-10 datasets respectively and present the experimental results under the 10-shot setting. Figure 7a–c show examples from the DIOR dataset, including airplane (novel class), tennis court (novel class), and storage tank (base class), respectively. Figure 7d–e show examples from the NWPU VHR-10 dataset, including baseball diamond (novel class) and harbor (base class), respectively.

Although MAML provides a good initialization for generalization, it is not well-suited for direct application to few-shot object detection tasks, as it lacks sufficient adaptability under limited supervision, resulting in missed detections and false positives. Fine-Tuning performs well in identifying novel classes, but it tends to overfit under limited data. Compared with Fine-Tuning, TFA adopts a two-stage framework that is more appropriate for few-shot learning and can detect most objects effectively but still struggles with certain classes, such as harbor against complex backgrounds and airplane with large pose variations. Both BC-YOLO* and BC-YOLO maintain stable performance on base classes, yet they fail to detect novel classes in scenarios involving densely distributed targets or objects with blurred boundaries. AOFS performs well on novel classes, mainly due to the strong geometric prior provided by its angle annotation. However, on the base class, its performance is slightly inferior to that of LFODet. In contrast, the proposed method achieves more satisfactory detection results while offering distinct advantages in handling densely distributed targets, maintaining robustness, and achieving balanced recognition across both novel and base classes. In summary, LFODet performs well in data-scarce scenarios, demonstrating strong multi-category discrimination on DIOR and effective multi-scale fusion on the densely distributed small-object NWPU VHR-10 dataset. It not only performs well in detecting novel classes but also maintains the detection of base classes, demonstrating decent accuracy and robustness, making it a reliable solution for few-shot object detection.

### 4.5. Ablation Experiments and Discussion

#### 4.5.1. Impact of GSA, GCA, and Fusion

To comprehensively validate the effectiveness of key modules within LFODet, i.e., GSA, GCA, and fusion, we conducted ablation experiments on the DIOR and NWPU VHR-10 datasets. The results are based on the first stage, Base Training.

As shown in Table 4, each module contributes differently to the final performance. GCA mainly improves channel-wise semantic representation by emphasizing informative feature channels. GSA mainly enhances fine-grained spatial feature modeling, which is important for small and densely distributed remote sensing objects. The feature fusion module integrates multi-level information from different network layers, thereby balancing high-level semantic information and low-level spatial details. Removing any of these modules causes a performance drop, while the full model achieves the best performance, demonstrating that these components are complementary rather than redundant.

Table 5 further reports the practical inference efficiency of different module combinations. The reported detection time includes not only model forward propagation but also preprocessing, bounding-box decoding, and NMS. Therefore, removing attention or fusion modules does not necessarily lead to faster end-to-end inference. When feature representation is weakened, the detector may generate more redundant, overlapping, or low-quality candidate boxes, which increases the cost of postprocessing, especially NMS. In contrast, the complete LFODet model improves semantic and spatial feature representation through GCA and GSA and further enhances multi-scale feature integration through the fusion module. As a result, it can produce more reliable candidate boxes and maintain favorable practical inference efficiency while improving detection performance.

#### 4.5.2. Impact of the Two Parallel Branches and Adding Base Data

**Figure 8:**
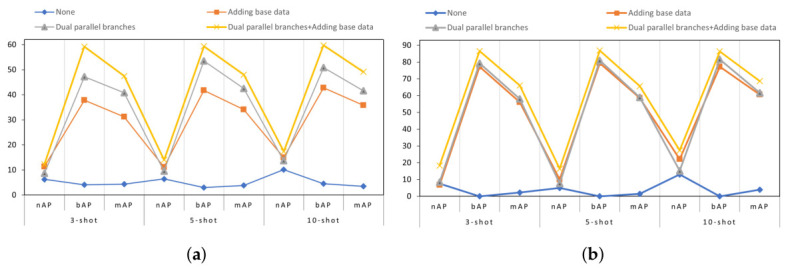
Ablation result comparison. (**a**) DIOR dataset. (**b**) NWPU VHR-10 dataset. Note: Data come from the mean.

After obtaining the optimized feature extraction network, as shown in Table 6, both parallel branches and the addition of base-class data during Fine-Tuning contribute to the final performance. Adding base-class data increases the diversity of training categories and reduces overfitting to limited novel-class samples. The two parallel branches help maintain base-class detection performance during inference while allowing the novel branch to adapt to few-shot novel classes. In this dual-branch inference strategy, the gating mechanism serves as a rule-based branch selection strategy, which compares the product of the classification score and objectness confidence score from the base and novel branches and retains the branch with the higher score as the final output. Therefore, the ablation results evaluate the overall effect of the dual-branch inference design rather than the gating mechanism alone. When both components are removed, the model performance drops sharply across all shot settings, highlighting their necessity. The best results are obtained when both components are used together, indicating their complementary effects. Figure 8 shows base data and how the two parallel branches play increasingly important roles.

### 4.6. Evaluation of Model Computational Efficiency

Considering the constraints on computation and memory, we evaluated several backbone networks (e.g., MobileNetv3, MobileNetv2, ShuffleNetv2, and GhostNetv2) and different convolution operations in the neck (e.g., Vanilla convolution, Depthwise Separable Convolution (DSC), and Ghost convolution). It should be noted that the evaluations in Table 4 and Table 7 are both conducted during the first stage, Base Training, using the fixed base-class training set. Therefore, these experiments aim to evaluate the contribution of architectural components and compare the architectural efficiency and representation capability of different combinations of backbone networks and convolution operations under the same Base Training protocol. In contrast, the third-stage few-shot Fine-Tuning experiments are repeated over five random few-shot splits to reduce the influence of support-set sampling randomness, thereby providing more reliable performance estimates.

As evidenced by the results in Table 7 (derived from the Base Training stage), the integration of Ghost convolution in the neck with MobileNetv3 backbone achieves the most outstanding performance. Specifically, when adopting MobileNetv3 as the backbone and applying Ghost convolution in the neck, the model provides a good balance between detection accuracy and computational efficiency. On the DIOR dataset, this configuration achieves the highest mAP while significantly reducing both FLOPs and Params compared with Vanilla convolution and DSC. Although DSC achieves slightly higher accuracy on the NWPU VHR-10 dataset, it comes at the cost of increased computation and parameters. Furthermore, under the same Ghost convolution setup, MobileNetv3 outperforms other lightweight backbones, i.e., MobileNetv2, ShuffleNetv2, and GhostNetv2, in overall balance, offering better mAP with lower computational overhead. Notably, ShuffleNetv2 incurs the highest FLOPs without a clear accuracy advantage, and GhostNetv2 performs the worst in detection accuracy despite its low complexity. These comparisons confirm that pairing MobileNetv3 with Ghost convolution is an effective strategy for achieving good detection results with limited computing resources. In addition, one complete three-stage LFODet training pipeline, including Base Training, Meta-Training, and 10-shot Fine-Tuning, requires approximately 36 h on the NVIDIA GeForce RTX 3060 GPU platform.

### 4.7. Discussion

Although LFODet has shown good performance on standard benchmark datasets, its feasibility in real-world applications largely depends on the model’s generalization ability in cross-domain and cross-sensor scenarios.

The primary challenge lies in cross-domain generalization, as the DIOR and NWPU VHR-10 datasets mainly consist of satellite images collected in specific lighting, seasonal, and geographic environments. Furthermore, the scale of these benchmarks and the narrow scope of evaluated object categories limit the comprehensiveness of our current evaluation. In practical deployment, models often need to process images from different geographical regions with distinct landscape features, which leads to significant domain shift issues. The lightweight design of our backbone network MobileNetv3 may not be as robust as large deep networks in capturing such cross-domain-invariant features.

Another key challenge stems from cross-sensor differences. Remote sensing data may originate from different satellite sensors or drone platforms, which exhibit significant variations in spatial resolution, imaging angles, and signal-to-noise ratio (SNR). Although the lightweight modules designed in our model improve computational efficiency, their adaptability to drastic changes in physical properties still requires further verification. In future work, physics-guided blocks [48] will be introduced into the network to boost detection performance.

To enhance the generalization ability of LFODet in practical applications, we will incorporate domain adaptation techniques into the training process in future research. This enables the model to learn domain-invariant features, which helps narrow the distribution gap between the source domain and target domain. Additionally, to comprehensively evaluate the model’s practical performance, we will utilize more diverse datasets and real-world scenarios and conduct more in-depth ablation studies in future work.

In summary, although the proposed model, LFODet, in this study has achieved promising results in few-shot detection tasks on specific datasets, its generalization ability in open environments remains a key focus for advancing toward practical applications. Specifically, addressing the aforementioned cross-domain and cross-sensor generalization capabilities will be critical to promoting the maturity of lightweight few-shot remote sensing detection technology. Additionally, in terms of computational complexity, compared with end-to-end models, the training and inference complexity of LFODet represents a necessary trade-off to achieve excellent performance within a limited parameter budget.

## 5. Conclusions

LFODet, a lightweight object detection model with meta-learning, is proposed to address few-shot challenges in remote sensing images in this paper. To enhance semantic and spatial fine-grained features, We integrate GCA and GSA modules into LFODet to enhance semantic and fine-grained spatial features. By leveraging the meta-learning strategy, LFODet can rapidly adapt to novel classes using limited samples. Moreover, during the inference phase, the two parallel branches equipped with the gating mechanism effectively maintain the recognition performance for base classes. The experiments on the DIOR dataset demonstrate LFODet’s satisfactory performance in few-shot scenarios. It not only achieves accurate recognition of novel classes but also preserves the detection performance for base classes. Ablation experiments further validate the effectiveness of the key components. The combination of computational efficiency and adaptation capability, as demonstrated on the DIOR and NWPU VHR-10 datasets, makes LFODet suitable for resource-constrained computing applications in the field of remote sensing.

In summary, this work demonstrates three key strengths of LFODet: (1) lightweight yet accurate detection, enabled by an efficient architecture design that maintains competitive performance with reduced computational cost; (2) rapid adaptability to novel classes, achieved by integrating meta-learning with dedicated attention mechanisms; (3) balanced performance on base and novel classes, realized through a dual-branch inference strategy.

## Figures and Tables

**Table 1:** Training configurations.

Setting	Base Training	Meta-Training	Fine-Tuning
Scheduler	Cosine decay	–	–
Optimizer	Adam	Adam	Adam
Learning rate	1.5 × 10^−4^ initial/ 1 × 10^−6^	1 × 10^−3^	8 × 10^−3^
Epoch	120	3	400
Batch size	10	4	16

Note: ‘–’ means none at this position.

**Table 2:** Experimental results on the NWPU VHR-10 and DIOR datasets.

Method	Backbone	Shots	DIOR				NWPU VHR-10		
			nAP	bAP	mAP		nAP	bAP	mAP
MAML [45]	MobileNetv3	3	0.91±0.40	18.32±0.62	13.97±0.42		0.57±0.90	4.04±2.52	3.00±1.73
		5	0.84±0.28	22.03±1.12	16.73±0.83		1.58±1.99	4.31±1.41	3.49±1.24
		10	1.06±0.06	23.10±1.03	17.59±0.77		0.50±0.42	3.20±4.31	2.39±3.02
Fine-Tuning [18]	MobileNetv3	3	5.74±1.97	25.08±3.42	20.25±2.94		20.78±6.55	3.34±2.72	8.57±2.08
		5	8.39±1.52	19.35±1.24	16.61±1.21		34.34±6.92	0.13±0.19	10.39±2.18
		10	12.23±1.32	9.05±5.87	9.85±4.15		45.04±2.28	0.00±0.00	13.51±0.68
TFA [46]	MobileNetv3	3	9.38±1.34	32.10±0.85	26.42±0.80		19.58±3.20	51.40±9.69	41.85±7.45
		5	11.37±1.03	33.02±0.83	27.61±0.72		34.81±7.86	57.21±4.49	50.49±3.64
		10	17.75±1.61	28.39±2.31	25.73±1.71		45.20±4.88	57.18±3.42	53.58±3.00
BC-YOLO * [41]	MobileNetv3	3	2.78±1.96	59.55±0.24	45.36±0.62		0.00±0.00	87.17±0.79	61.02±0.55
		5	3.76±1.06	59.91±0.22	45.87±0.42		0.00±0.00	88.14±0.76	61.70±0.53
		10	8.18±2.17	59.92±0.12	46.98±0.59		0.00±0.00	86.96±0.70	60.87±0.49
BC-YOLO [41]	Darknet-53	3	1.52±0.11	38.58±0.36	29.31±0.29		0.09±0.13	81.86±1.83	57.33±1.29
		5	2.16±0.46	37.58±1.34	28.73±1.07		1.10±0.96	82.82±2.68	58.30±1.92
		10	4.81±0.66	38.59±0.77	30.14±0.50		2.91±0.72	84.94±2.03	60.33±1.57
AOFS [47]	CSPDarknet-53	3	18.50±4.58	8.02±1.28	10.64±0.29		39.78±4.22	32.85±2.21	34.92±1.96
		5	19.30±4.17	11.29±0.81	13.30±1.01		43.13±9.12	40.49±4.09	39.84±3.81
		10	24.27±4.42	15.81±0.34	17.40±1.01		52.97±7.07	51.10±5.11	48.88±9.02
LFODet	MobileNetv3	3	12.98±1.33	59.52±0.24	47.31±0.68		11.43±6.54	87.08±0.34	64.38±2.08
		5	14.38±1.80	59.56±0.41	47.91±0.56		7.20±2.26	88.46±0.87	64.08±0.98
		10	16.47±1.33	59.73±0.35	48.39±0.77		24.24±4.61	87.47±0.58	68.50±1.66

Note: Bold font indicates the best results, and underlining indicates the second-best results within each dataset. The results of LFODet are based on the third stage, Fine-Tuning. The DIOR dataset results follow the same experimental protocol as NWPU VHR-10. * denotes the BC-YOLO variant using MobileNetV3 as the backbone.

**Table 3:** Comparison of the experimental results of novel classes on DIOR.

Method	Shot	Airplane	Baseball Field	Tennis Court	Train Station	Wind Mill	nAP
MAML [45]	3	0.01±0.02	4.20±1.58	0.28±0.52	0.01±0.03	0.05±0.03	0.91±0.40
	5	0.02±0.01	4.05±1.39	0.07±0.05	0.06±0.05	0.02±0.01	0.84±0.28
	10	0.03±0.02	5.06±0.34	0.07±0.09	0.10±0.06	0.03±0.01	1.06±0.06
Fine-Tuning [18]	3	0.38±0.30	12.00±6.44	5.53±4.26	2.72±2.46	8.04±4.43	5.74±1.97
	5	2.02±1.40	9.82±6.16	20.14±6.49	1.78±0.88	8.21±4.26	8.39±1.52
	10	3.05±2.34	18.29±7.25	18.56±12.16	5.87±1.13	15.40±5.09	12.23±1.32
TFA [46]	3	1.26±0.80	18.17±9.64	12.18±8.89	3.67±2.96	11.60±2.50	9.38±1.34
	5	2.85±2.06	18.23±9.28	20.30±8.09	4.19±3.59	11.30±5.08	11.37±1.03
	10	7.01±3.95	32.70±6.28	20.43±8.95	7.94±2.98	20.69±4.92	17.75±1.61
BC-YOLO * [41]	3	0.15±0.18	12.56±9.90	0.02±0.03	1.05±1.47	0.13±0.10	2.78±1.96
	5	0.38±0.41	16.05±4.41	0.34±0.35	1.69±1.01	0.23±0.21	3.76±1.06
	10	0.56±0.34	33.56±7.32	3.50±2.11	2.94±2.15	0.34±0.11	8.18±2.17
BC-YOLO [41]	3	0.04±0.06	5.61±0.55	0.66±0.36	1.17±0.52	0.11±0.07	1.52±0.11
	5	0.66±0.40	7.42±1.70	1.16±0.53	1.53±0.15	0.01±0.02	2.16±0.46
	10	0.94±0.09	12.85±2.02	3.04±1.50	4.98±1.89	2.23±1.61	4.81±0.66
AOFS [47]	3	17.80±9.18	27.54±23.59	27.43±12.34	1.03±0.75	11.29±2.10	18.50±4.58
	5	18.23±6.34	39.60±9.96	25.89±15.21	1.92±1.25	10.89±2.94	19.30±4.17
	10	21.33±8.66	43.83±7.67	40.60±14.84	2.87±0.68	12.71±5.05	24.27±4.42
LFODet	3	2.25±1.51	35.31±3.80	20.40±6.64	3.72±2.14	3.24±0.74	12.98±1.33
	5	4.15±1.72	39.20±9.27	22.33±6.52	4.46±3.13	1.79±0.58	14.38±1.80
	10	3.96±0.73	39.75±9.02	24.49±9.01	4.70±2.74	6.15±1.68	16.47±1.33

Note: Bold font indicates the best results, and underlining indicates the second-best results. AP is computed at an IoU threshold of 0.5. The results are averaged over three independent random few-shot splits. * denotes the BC-YOLO variant using MobileNetV3 as the backbone.

**Table 4:** Ablation experiments and evaluations of LFODet on feature extraction network.

Methods	Base Training	GSA	GCA	Fusion	mAP (DIOR)	mAP (NWPU VHR-10)	FLOPs (G)	Params (M)
Selected Module(s)	✓	✓	✓	✓	67.5	89.6	9.654	10.06
	✓		✓	✓	60.5 (−7.0)	80.4 (−9.2)	8.661 (−0.993)	8.85 (−1.21)
	✓	✓		✓	59.6 (−7.9)	84.1 (−5.5)	9.144 (−0.510)	7.44 (−2.62)
	✓	✓	✓		64.4 (−3.1)	82.4 (−7.2)	7.708 (−1.946)	9.37 (−0.69)
	✓				57.9 (−9.6)	75.3 (−14.3)	4.279 (−5.375)	4.24 (−5.82)

Note: “✓” indicates that the corresponding component is used. The values in parentheses denote the changes in each metric relative to the complete LFODet model. This ablation study is conducted during the first stage, Base Training, using the fixed base-class training set. Therefore, the results are reported under the same Base Training protocol rather than over multiple few-shot random splits.

**Table 5:** Ablation study of the proposed modules and inference efficiency of LFODet on the NWPU VHR-10 dataset.

Methods	Base Training	GSA	GCA	Fusion	FPS	Detection Time (ms/img)
Selected Module(s)	✓	✓	✓	✓	9.66	103.55
	✓		✓	✓	7.27	137.55
	✓	✓		✓	7.35	135.97
	✓	✓	✓		5.64	177.30
	✓				4.83	207.02

Note: “✓” indicates that the corresponding component is used. All results are obtained under the first-stage setting, Base Training, using the same training and evaluation protocol. FPS is calculated as 1000 divided by the average detection time per image. The detection time includes image preprocessing, model forward propagation, bounding-box decoding, and NMS.

**Table 6:** Ablation experiments and evaluations of the proposed LFODet.

Shots	Parallel Branches	Adding Base Data	DIOR				NWPU VHR-10		
			nAP	bAP	mAP		nAP	bAP	mAP
3	✓	✓	12.24±0.97	59.23±0.16	47.48±0.16		18.26±2.59	86.59±0.47	66.09±1.10
	✓		8.67±0.81	47.22±4.98	40.80±4.21		8.46±2.08	79.27±0.62	58.03±0.37
		✓	11.36±1.12	37.82±0.98	31.21±0.84		6.75±1.00	77.20±2.42	56.08±1.99
			6.25±0.66	4.07±0.23	4.32±0.49		7.57±1.55	0.00±0.00	2.26±0.46
5	✓	✓	14.02±0.38	59.34±0.34	48.01±0.26		16.40±1.30	86.89±0.66	65.75±0.67
	✓		9.50±1.16	53.50±4.42	42.50±3.06		7.62±0.57	80.99±0.13	58.98±0.25
		✓	11.15±0.80	41.73±2.22	34.09±1.86		10.00±0.91	79.40±2.57	58.58±1.97
			6.43±0.14	2.98±0.58	3.85±0.45		4.96±0.35	0.00±0.00	1.48±0.11
10	✓	✓	17.36±0.52	59.74±0.21	49.15±0.28		27.33±1.27	86.35±0.72	68.65±0.16
	✓		13.72±1.01	50.86±7.13	41.57±5.49		15.20±2.46	81.61±0.66	61.69±1.11
		✓	14.97±1.06	42.77±3.48	35.82±2.87		22.12±0.97	77.22±0.68	60.70±0.37
			10.16±0.38	4.49±2.61	3.47±0.06		13.00±0.74	0.00±0.00	3.90±0.22

Note: “✓” indicates that the corresponding component is used. The results are based on the third stage, Fine-Tuning.

**Table 7:** Evaluation of LFODet with different backbone networks and convolution operations.

Backbone	Vanilla	DSC	Ghost	mAP (DIOR)	mAP (NWPU VHR-10)	FLOPs (G)	Params (M)
MobileNetv3	✓			62.5	75.2	14.555	14.87
		✓		63.2	92.2	14.735	14.98
			✓	67.5	89.6	9.654	10.06
MobileNetv2			✓	63.1	81.8	12.153	12.71
ShuffleNetv2			✓	62.4	86.5	22.377	13.80
GhostNetv2			✓	53.6	37.8	11.293	13.30

Note:“Vanilla”, “DSC”, and “Ghost” denote the convolution operations used in the neck. “✓” indicates that the corresponding component is used. The input feature-map channels of the three neck branches are 40/112/960 for MobileNetV3, 32/96/1280 for MobileNetV2, 176/352/1024 for ShuffleNetV2, and 48/136/1152 for GhostNetV2. The results are reported under the same Base Training protocol rather than over multiple few-shot random splits.

## Data Availability

Data are available at the following URLs: Google Drive: https://drive.google.com/open?id=1UdlgHk49iu6WpcJ5467iT-UqNPpx__CC (accessed on 6 July 2026); OneDrive: https://1drv.ms/u/s!AmgKYzARBl5cczaUNysmiFRH4eE (accessed on 6 July 2026).
